# Supplementary figures and images for: GCL loss in BRAO
Source: PLoS One. 2023 Jan 5;18(1):e0279920. doi: 10.1371/journal.pone.0279920 (PMC9815566; doi:10.1371/journal.pone.0279920)

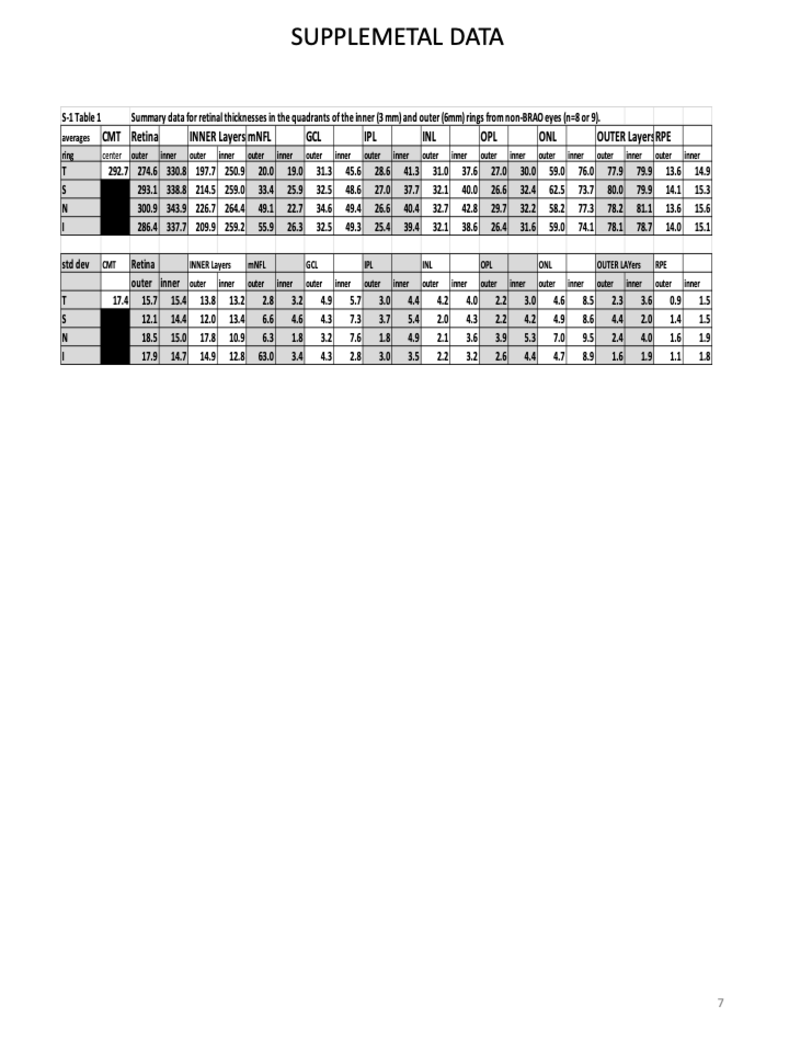

Supplement: S1 Table — (TIF) [file pone.0279920.s001.tif]

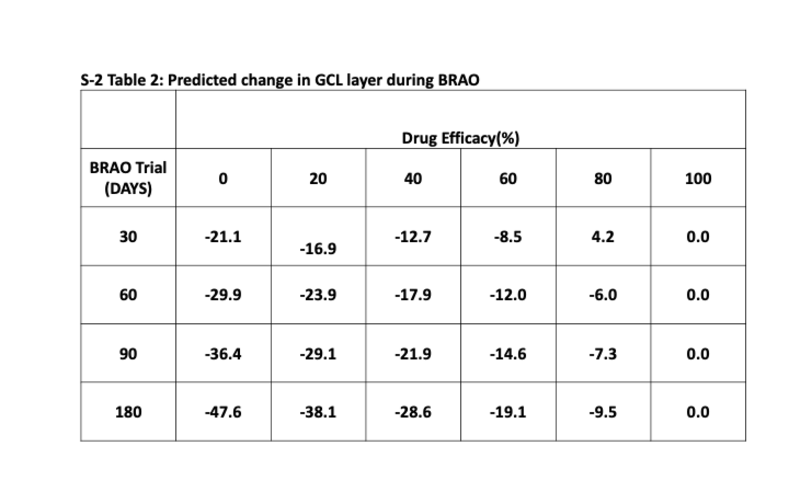

Supplement: S2 Table — (TIF) [file pone.0279920.s002.tif]

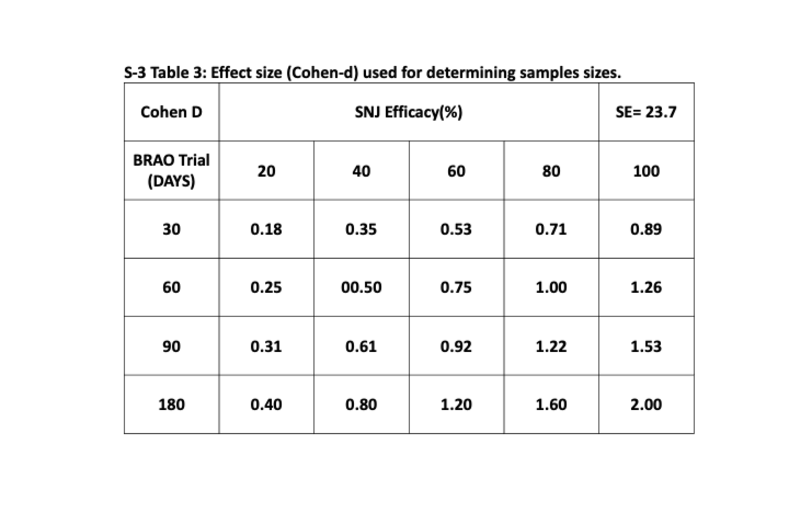

Supplement: S3 Table — (TIF) [file pone.0279920.s003.tif]
