# Supplementary material for: GCL loss in BRAO
Source: PLoS One. 2023 Jan 5;18(1):e0279920. doi: 10.1371/journal.pone.0279920 (PMC9815566; doi:10.1371/journal.pone.0279920)
Supplement: Attachment — Submitted filename: PONE-D-22-15899_comments.docx [file pone.0279920.s004.docx]

Based on their previous study which showed OCT could be used to follow thinning of the GCL in CRAO, the authors here found that the thickness of the GCL layer in BRAO decreased rapidly with time as in CRAO, and GCL thickness could be used to provide sample size statistics in a clinical trial to test a calpain inhibitor.

1. In ‘Introduction’, since ‘the purpose of the studies described below was to use OCT to follow GCL thinning in BRAO and to predict the number of patients and observations times needed for clinical trials of SNJ1945 against BRAO’, it would be better to add some information about the patients number prediction and observation times for clinical trials from other researches.

2. At the end of the ‘Materials’, last sentence, add ‘.’ and the software you used.

3. In ‘Results-Demographics’, since the ‘co-morbidity factor’ existed, it would be better to be discussed in the discussion.
