# Supplementary material for: GCL loss in BRAO
Source: PLoS One. 2023 Jan 5;18(1):e0279920. doi: 10.1371/journal.pone.0279920 (PMC9815566; doi:10.1371/journal.pone.0279920)
Supplement: Attachment — Submitted filename: Response to Reviewers.docx [file pone.0279920.s005.docx]

1. Reviewer 1 comment: …… it would be better to add some information about the patient number prediction and observation times for clinical trials from other researches. Author response and revision: Last sentence presents this and is newly referenced.
2. Reviewer 1 comment: At the end of the ‘Materials’, last sentence, add ‘.’ and the software you used. Author response and revision: The “.” was added at the end of the sentence, and the software was indicated in the middle of the sentence.
3. Reviewer 1 comment: In ‘Results-Demographics’, since the ‘co-morbidity factor’ existed, it would be better to be discussed in the discussion. Author response and revision: As requested the co-morbidity sentence was removed from the “Demographics” section and moved to the 3^rd^ paragraph of the Discussion.

Additional journal requirements:

4. Style requirements

The entire revised manuscript was updated to PLOS ONE formatting style requirements

5. Updated funding statement. “I have read the journal's policy and the authors of this manuscript have the following competing interests: TRS, contract SRA-17-092, Senju Pharmaceutical Co. Ltd. MA is an employee of Senju. MP, TS and PS are paid consultants. The funder provided support in the form of salary for author [MA], but did not have any additional role in the study design, data collection and analysis, decision to publish, or preparation of the manuscript. The specific roles of the authors are articulated in the ‘author contributions’ section. This does not alter our adherence to  PLOS ONE policies on sharing data and materials.”
6. Review your reference list to ensure that it is complete and correct, indicating changes.

References were updated and conform to style requirements.

Uploaded three files labeled:

“Response to Reviewers.”

“Revised Manuscript with Track Changes,” The marked-up manuscript highlighting changes made to the original version labeled

“Manuscript,” the unmarked revised manuscript.
